# Myoblasts With Higher IRS-1 Levels Are Eliminated From the Normal Cell Layer During Differentiation

**DOI:** 10.3389/fendo.2020.00096

**Published:** 2020-02-28

**Authors:** Ryosuke Okino, Ami Usui, Yosuke Yoneyama, Shin-Ichiro Takahashi, Fumihiko Hakuno

**Affiliations:** ^1^Departments of Animal Sciences and Applied Biological Chemistry, Graduate School of Agriculture and Life Sciences, The University of Tokyo, Tokyo, Japan; ^2^Laboratory of Cell Regulation, Departments of Animal Sciences and Applied Biological Chemistry, Graduate School of Agriculture and Life Sciences, The University of Tokyo, Tokyo, Japan

**Keywords:** insulin-like growth factor (IGF)-I, L6 myoblasts, myogenesis, insulin receptor substrate (IRS)-1, cell competition

## Abstract

Insulin receptor substrate (IRS)-1 is a major substrate of insulin-like growth factor (IGF)-I receptors. It is well-known that IGF-I and II play essential roles in myogenesis progression. Herein, we report an unexpected phenomenon that IRS-1-overexpressing L6 myoblasts are eliminated from normal cell layers at the beginning of differentiation. Initially, the IRS protein level and apoptosis were examined during myogenic differentiation in L6 myoblasts. We found that the IRS-1 protein level decreased, whereas active caspase 3 increased around 1 day after induction of differentiation. The addition of a pan-caspase inhibitor, Z-VAD-FMK, inhibited differentiation-induced suppression of the IRS-1 protein level. Apoptosis was not enhanced in L6 myoblasts stably expressing high levels of IRS-1 (L6-IRS-1). However, when L6-IRS-1 was cultured with control cells (L6-mock), we observed that L6-IRS-1 was eliminated from the cell layer. We have recently reported that, in L6-IRS-1, internalization of the IGF-I receptor was delayed and IGF signal activation was sustained for a longer period than in L6-mock. When cells stably expressing IRS-1 3YA mutant, which could not maintain the IGF signals, were cultured with normal cells, elimination from the cell layer was not detected. These data suggested that the high level of IRS-1 in myoblasts induces elimination from the cell layer due to abnormal sustainment of IGF-I receptor activation.

## Introduction

Myogenic differentiation is a tightly regulated complex process in which mononucleated myoblasts proliferate, express myogenic marker proteins (MyoD, myogenin, myosin heavy chain (MyHC), etc.), and fuse to form multinucleated myotubes. Matured myotubes convert into myofibers, which are capable of muscle contraction. These multiple processes of myogenic differentiation seem to depend on numerous pathways (1, 2). Extensive investigations using myoblast cell lines and tissues revealed that several extracellular growth factors modulate myogenic differentiation (3–5). Many papers have shown that insulin and insulin-like growth factors (IGFs) stimulate myoblast differentiation and are required for skeletal muscle development (6–9).

IGF-I and IGF-II are anabolic hormones with structures similar to that of proinsulin. IGFs are revealed to possess various bioactivities, including the induction of cell proliferation, differentiation, and survival of target tissues. Generally, by binding to their specific receptors on the plasma membrane, IGFs activate intrinsic tyrosine kinase activity. The activated receptor phosphorylates several substrates, including insulin receptor substrates (IRSs). Phosphotyrosine residues in IRSs are recognized by several signaling molecules with an SH2 domain, resulting in activation of the phosphatidyl inositol 3-kinase-Akt pathway and Ras-mitogen activated protein kinase pathway. Activation of these pathways is shown to be required for the expression of various IGF bioactivities.

It is well-established that IGFs are required for myogenic differentiation. In particular, in serum-free medium, myogenic differentiation was blocked and IGF addition significantly enhanced the creatine kinase level (10). Thus, IGF has an essential role in myogenic differentiation. However, it is unclear whether the activation of downstream IGF signaling pathways is constantly required for myogenesis. For example, IRS-1 knockdown C2C12 myoblasts had defects in myogenesis (11). On the other hand, we previously reported that IRS-1 overexpression inhibited myogenic differentiation in L6 myoblasts through continuous Foxo1 inhibition that might cause repression of MyHC at the late stage of differentiation (12).

Recent reports demonstrated that apoptotic cells are necessary for the myogenic differentiation process. The phosphatidylserine receptor BAI1, which was previously linked to apoptotic cell recognition by phagocytes, promotes myoblast fusion. Blocking apoptosis during myogenic differentiation potently impaired this process; furthermore, returning apoptotic myoblasts to this system restored fusion (13). On the other hand, endoplasmic reticulum (ER) stress signaling occurs during myoblast differentiation, and inhibition of ER stress signaling blocked apoptosis and myoblast differentiation. Moreover, increased ER stress enhanced differentiation-associated apoptosis of myoblasts (14). Thus, apoptosis is required for myogenic differentiation. However, the types of cells that selectively undergo apoptosis or differentiate into myotubes during myogenic differentiation remain unknown.

This study was undertaken to evaluate the mechanism of IGF signal regulation of myoblast proliferation and apoptosis during myogenic differentiation. We found that cells expressing high IRS-1 levels are eliminated from the normal cell layer and undergo apoptosis upon culturing with normal cells due to sustained IGF signal activation.

## Materials and Methods

### Materials

Dulbecco's modified Eagle's medium (DMEM) was purchased from Nissui Pharmaceutical Co. (Tokyo, Japan). Fetal bovine serum (FBS) was obtained from Sigma Aldrich (St. Louis, MO, USA). Penicillin and streptomycin were obtained from Banyu Pharmaceutical Co. (Ibaraki, Japan). Z-VAD-FMK was obtained from BD Biosciences (New York, NY, USA).

### Antibodies

Anti-IRS-2 (390761) antibody was obtained from Santa Cruz Biotechnology, Inc. (Santa Cruz, CA, USA). Anti-IRS1 (06-248), anti-myosin heavy chain (05-716) and anti-p85 (06-195) antibodies were acquired from Millipore (Billerica, MA, USA). Anti-caspase 3 (#9662), anti-cleaved caspase 3 (#9661), and anti-Bax (#2772) antibodies were purchased from Cell Signaling Technology, Inc. (Danvers, MA, USA). Horseradish peroxidase (HRP)-conjugated secondary anti-rabbit (NA934) and anti-mouse IgG (NA931) antibodies were obtained from GE Healthcare (Pittsburgh, PA, USA). Antibodies were diluted according to the recommendations on their data sheets. Enhanced chemiluminescence (ECL) reagents were acquired from PerkinElmer Life Science (Boston, MA, USA). Alexa Fluor 488 or 594-conjugated secondary anti-mouse, anti-rabbit, or anti-rat IgG antibodies were obtained from Invitrogen (Carlsbad, CA, USA).

### Cell Culture

L6 cells were maintained at 37°C in a humidified 5% CO_2_-controlled atmosphere in DMEM supplemented with 10% FBS, 0.1% NaHCO_3_, 50 IU/mL penicillin, and 50 μg/mL streptomycin. L6 cell differentiation was induced as previously described (12). Passage number of cells used in experiments was 8~15. In each experiment, passage number of the cell lines are same. PLAT-E cells were cultured for retrovirus packaging as previously described (15).

### Retrovirus Production and Generation of Stable Cell Lines

We generated the constructs of the pMX-neo vectors containing IRS-1 (pMX-GFP-IRS-1, pMX-mycIRS-1, and pMX-IRS-1-3YA) and pMX-puro vector containing GFP (pMX-GFP). Retrovirus production and transduction in L6 cells were performed as described previously (15). Briefly, PLAT-E cells (provided by T. Kitamura, The University of Tokyo, Tokyo, Japan) were transiently transfected with each pMX vector using polyethylenimine (PEI) reagent, and the media containing the retrovirus were collected. L6 cells were incubated with the virus-containing medium supplemented with 2 mg/L polybrene. Uninfected cells were removed by G418 or puromycin selection. Isolation of the stable L6 line was performed as described previously (12).

### Cell Attachment Assay

Five million L6-mock cells were seeded on a 35 mm dish and cultured until confluent. Either L6-mycIRS-1-GFP or L6-GFP cells were then seeded on the L6-mock confluent cell layer or on a vacant dish. One day after the incubation, the cells were fixed, and the numbers of GFP-positive cells were counted. The cell layer attachment index (CLAI) was calculated as the number of GFP-positive cells attached on the cell layer divided by GFP-positive cells attached on the vacant dish.

### Immunoblotting

Cells were lysed at 4°C with ice-cold lysis buffer (1% NP40, 50 mM Tris-HCl [pH 7.4], 150 mM NaCl, 1 mM EDTA, 1 mM NaF, 10% glycerol, 20 μg/mL phenylmethylsulfonyl fluoride (PMSF), 5 μg/mL pepstatin, 10 μg/mL leupeptin, 100 KIU/mL aprotinin, 1 mM Na_3_VO_4_, and 10 mg/mL *p*-nitrophenyl phosphate), or ice-cold RIPA buffer (50 mM Tris-HCl [pH 7.4], 15 mM NaCl, 0.1% SDS, 0.5% deoxycholate, 20 μg/mL PMSF, 5 μg/mL pepstatin, 10 μg/mL leupeptin, 100 KIU/ml aprotinin, 1 mM Na_3_VO_4_, and 10 mg/mL *p*-nitrophenyl phosphate). Insoluble materials were removed by centrifugation at 15,000 × g for 10 min at 4°C, and the supernatant was prepared as a total cell lysate. Immunoblotting was performed as described previously (15).

### Immunofluorescence Staining

For confocal microscopy analysis, L6 cells were grown on coverslips. The cells were fixed for 10 min at 25°C in prewarmed 4% paraformaldehyde in phosphate-buffered saline (PBS). After washing three times with PBS, cells were permeabilized with 0.25% Triton X-100 in PBS at 25°C for 10 min. The cells were washed three times with PBS and then blocked for 1 h at 4°C with bovine serum albumin (BSA) blocking buffer (3% BSA and 0.025% NaN_3_ in PBS). Primary antibodies diluted in BSA blocking buffer were added overnight at 4°C. The samples were washed three times with PBS and incubated for 1 h at 25°C in a solution of Alexa Fluor-conjugated secondary antibodies diluted in BSA blocking buffer. The coverslips were mounted using Vectashield for visualization using a fluorescence microscope (KEYENCE, Tokyo, Japan) or confocal fluorescence microscope (OLYMPUS, Tokyo, Japan).

### Statistical Analysis

Statistical analyses of data were performed using Stat View software (Abacus Concepts, Inc., Berkeley, CA, USA). Comparisons between two groups were analyzed by Student's *t*-test, and more than two groups were analyzed by ANOVA followed by Turkey's test. Differences were considered to be statistically significant at *P* < 0.05, as represented by ^*^.

## Results

### Protein Levels of IRS-1 and Cleaved Caspase 3 Were Dramatically Changed During Myogenic Differentiation of L6 Myoblasts

**Figure 1:**
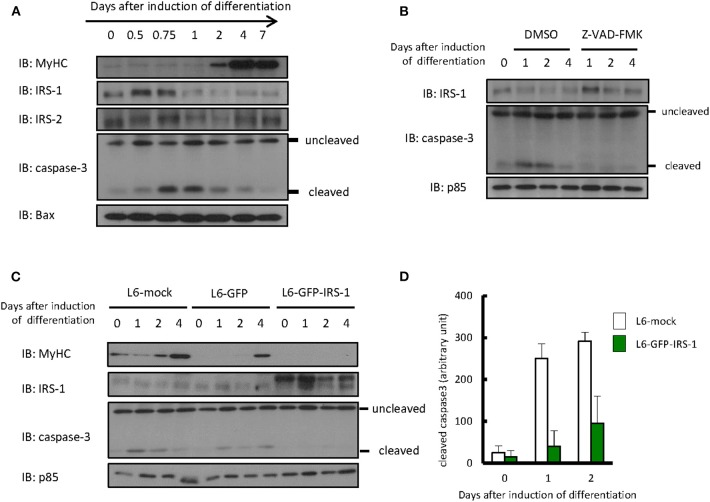
Protein level of IRSs and cleaved caspase 3 during myogenic differentiation of L6 myoblasts. **(A)** Differentiation of L6 myoblasts was induced by changing media from DMEM with 10% FBS to DMEM with 2% FBS. At the indicated days after differentiation induction, cell lysates were prepared, and total cell lysates were produced for immunoblotting analysis using the indicated antibodies. **(B)** Differentiation was induced in the differentiation medium with or without 100 μM Z-VAD-FMK. Immunoblotting was conducted using the indicated antibodies at the indicated days after differentiation induction. **(C)** L6-mock, L6-GFP, and L6-GFP-IRS-1 were induced to differentiate into myotubes. Immunoblotting was conducted at the indicated days after differentiation induction. **(D)** At the indicated days after differentiation induction, cells were fixed by PFA and immunostained with anti-cleaved caspase 3 antibody. The number of cleaved caspase 3-positive cells was counted, and the data is shown as means ± SEM. These are representative data from experiments independently performed twice.

Differentiation of L6 myoblasts was induced by changing media from DMEM with 10% FBS to DMEM with 2% FBS. As shown in Figure 1A, we could confirm that expression of the myogenic marker protein myosin heavy chain increased 2 days after the induction of differentiation. Protein levels of IRS-1 or IRS-2 were examined by immunoblotting analysis. The IRS-2 protein level was not changed during differentiation induction, whereas that of IRS-1 decreased only 1 day after induction. Interestingly, the level of cleaved caspase 3, an apoptotic marker protein and active form of caspase 3, increased ~0.75 day after differentiation induction; this indicated that apoptotic cells were generated, then IRS-1 protein was decreased. In addition, when the apoptosis inhibitor Z-VAD-FMK was added to the differentiation medium, the IRS-1 protein level did not decrease (Figure 1B). Since the IRS-1 protein level decreased just after apoptosis activation, we generated the hypothesis that cells highly expressing IRS-1 selectively undergo apoptosis.

To address whether IRS-1 overexpression enhances apoptosis, we infected L6 myoblasts with retroviruses expressing mock vector, GFP, or GFP-fused IRS-1 and isolated the stable cell lines L6-mock, L6-GFP, and L6-GFP-IRS-1. We could confirm that the GFP-IRS-1 expression level was high in L6-GFP-IRS-1 lines (Figure 1C). Caspase 3 activation was examined and found to be activated 1 day after inducing differentiation in L6-mock and L6-GFP control cells. However, in L6-GFP-IRS-1, caspase 3 was not activated (Figure 1C). Immunostaining analysis against cleaved caspase 3 (active caspase 3) also indicated that apoptosis was suppressed in L6-GFP-IRS-1 cells (Figure 1D). These data indicated that IRS-1 overexpression did not enhance apoptosis.

### Cells Overexpressing IRS-1 Were Selectively Excluded When They Were Surrounded by Normal Cells

**Figure 2:**
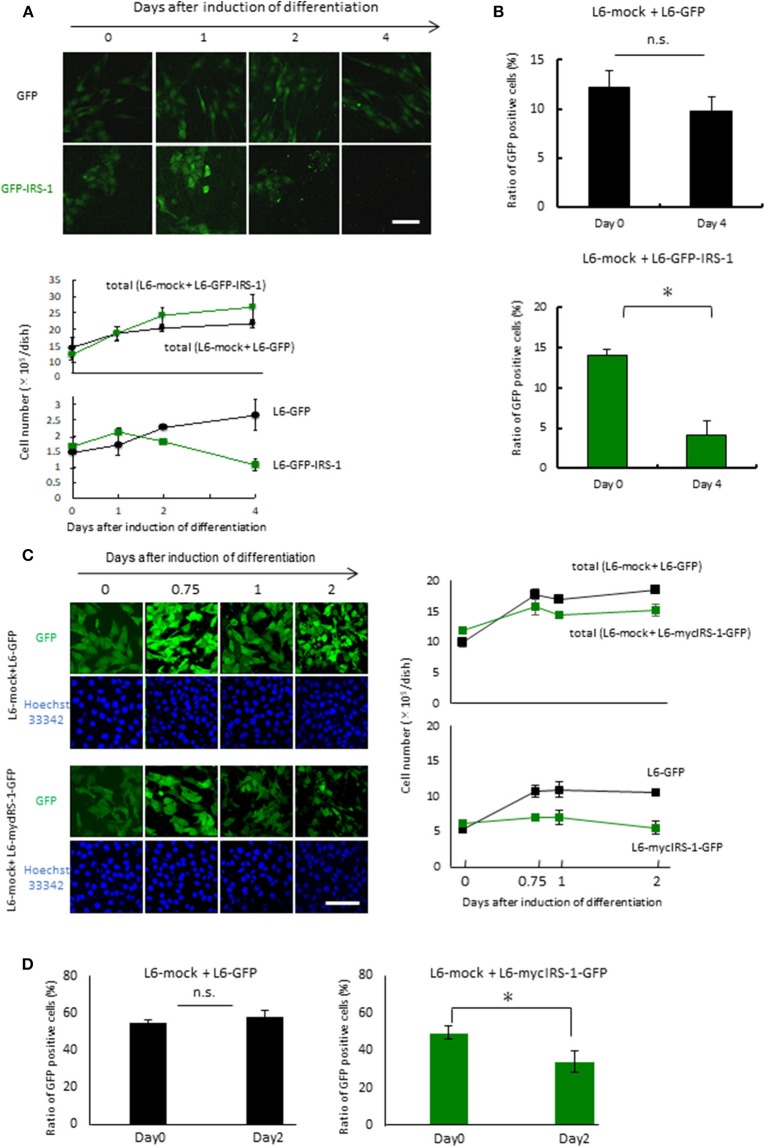
Elimination of cells highly expressing IRS-1. **(A)** Mixtures of L6-GFP and L6-mock or L6-GFP-IRS-1 and L6-mock were inoculated into dishes at a 1:10 ratio, and differentiation was induced. At the indicated days after differentiation induction, cells were fixed by PFA, and the total nucleus numbers and the nucleus numbers of GFP-positive cells were counted (right graphs). Scale bar: 100 μm. **(B)** The percentage of GFP-positive cells was calculated at day 0 or 4 after differentiation induction, as shown in the graph. Data is shown as means ± SEM. *p < 0.05 vs. day 0. **(C)** Mixtures of L6-GFP and L6-mock or L6-mycIRS-1-GFP and L6-mock were inoculated into the dishes at ratios of 1:1, and differentiation was induced. At the indicated days after differentiation induction, cells were fixed by PFA, and the numbers of GFP-positive and GFP-negative cells were counted (lower graphs). Scale bar: 100 μm. **(D)** The percentage of GFP-positive cells was calculated at day 0 or 4 after differentiation induction, as shown in the graph. Data is shown as means ± SEM. *p < 0.05 vs. day 0. These are representative data from experiments independently performed at least three times.

To examine the fate of cells overexpressing IRS-1 within a normal cell population, L6-GFP-IRS-1 or L6-GFP stable cell lines were mixed with normal L6 cells (L6-mock) at a ratio of 1:10. These cells were then cultured in 10% FBS medium until confluent. The mixture of the two cell lines was cultured in the differentiation medium for the indicated days. When L6-GFP was cultured with normal L6-mock, the number of GFP-positive cells (L6-GFP) increased at a similar ratio as that of the total cell number (Figure 2A). On the contrary, when L6-GFP-IRS-1 was cultured with L6-mock, the cell number of L6-GFP-IRS-1 decreased (Figure 2A). When L6-GFP was cultured with L6-mock, the ratio of GFP-positive cells remained unchanged until day 4 compared to day 0. However, when L6-GFP-IRS-1 was cultured with L6-mock, the ratio of GFP-positive cells decreased at day 4 compared to day 0 (Figure 2B). These data strongly suggested that L6-GFP-IRS-1 was selectively excluded from the cell layer.

Because protein degradation of IRS-1 is induced by the activation of the downstream IGF signal kinase mTORC1 (16–19), it is possible that the level of GFP-fused IRS-1 also degraded and the GFP signal diminished in response to IGF signal activation. To exclude this possibility, we generated stable cell lines expressing both mycIRS-1 and GFP independently (L6-mycIRS-1-GFP). At first Caspase 3 activation was examined in the single culture system. By the induction of differentiation, Caspase 3 activation was not enhanced also in L6-mycIRS-1-GFP (Figure S1). When L6-mycIRS-1-GFP was cultured with L6-mock at a ratio of 1:1, it was also selectively eliminated (Figure 2C). The ratio of L6-mycIRS-1-GFP cells decreased on day 2 compared to day 0, while the ratio of L6-GFP cells remained unchanged (Figure 2D). Moreover, growth rate of L6-mock, L6-GFP, or L6-mycIRS1-GFP was almost comparable (Figure S2). Although IRS-1 overexpression did neither enhance apoptosis (Figures 1C,D, Figure S1) nor suppress proliferation rate (Figure S2), the cell number of L6-mycIRS-1-GFP are selectively decreased only when these cells are cultured with normal cells (Figure 2). These data strongly suggested that cells highly expressing IRS-1 are selectively excluded from the cell layer.

### The Ability of Cells With Higher IRS-1 Levels to Attach to the Normal Cell Layer Was Impaired

**Figure 3:**
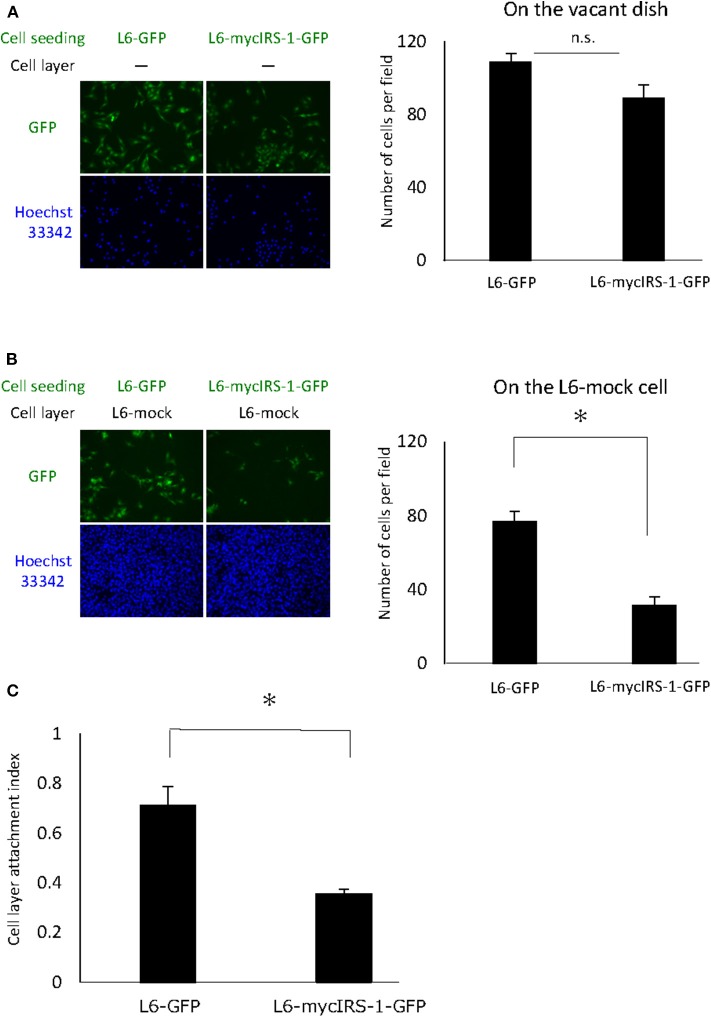
Attachment ability of cells highly expressing IRS-1. **(A)** Five million L6-mock cells were seeded on the dish and cultured until confluent. Either L6-mycIRS-1-GFP or L6-GFP was then seeded on the L6-mock confluent cell layer or directly on a dish. After 1 day of incubation, the cells were fixed and the numbers of GFP-positive cells were counted. **(B)** CLAI was defined as shown. **(C)** CLAI was calculated, and data are shown as means ± SEM (*n* = 3), *p < 0.05. These are representative data independently performed at least three times.

Cells with high IRS-1 levels were eliminated upon culturing with normal cells. These data suggested that cell-cell contact plays important roles in this phenomenon. Thus, we examined the cell attachment ability of cells with high IRS-1 levels. Initially, L6-mock cells were seeded on the dish and cultured until confluent; then, L6-mycIRS-1-GFP or L6-GFP cells were seeded on the confluent L6-mock cell layers. As a control, L6-mycIRS-1-GFP cells or L6-GFP cells were seeded on the vacant dishes. The number of cells attached to the vacant dishes was comparable between L6-GFP and L6-mycIRS-1-GFP (Figure 3A). However, the number of L6-mycIRS-1-GFP attached on the L6-mock layer was significantly lower than that of L6-GFP (Figure 3B). The ratio of the GFP-positive cell number on the L6-mock cell layers to the number of GFP-positive cells on the vacant dishes was defined as CLAI. As a result, the cell attachment ability of L6-mycIRS-1-GFP to L6-mock was significantly lower than that of L6-GFP to L6-mock cells (Figure 3C).

### Cells With Higher Levels of IRS-1 3YA Mutant Were Not Excluded Upon Culturing With Normal Cells

**Figure 4:**
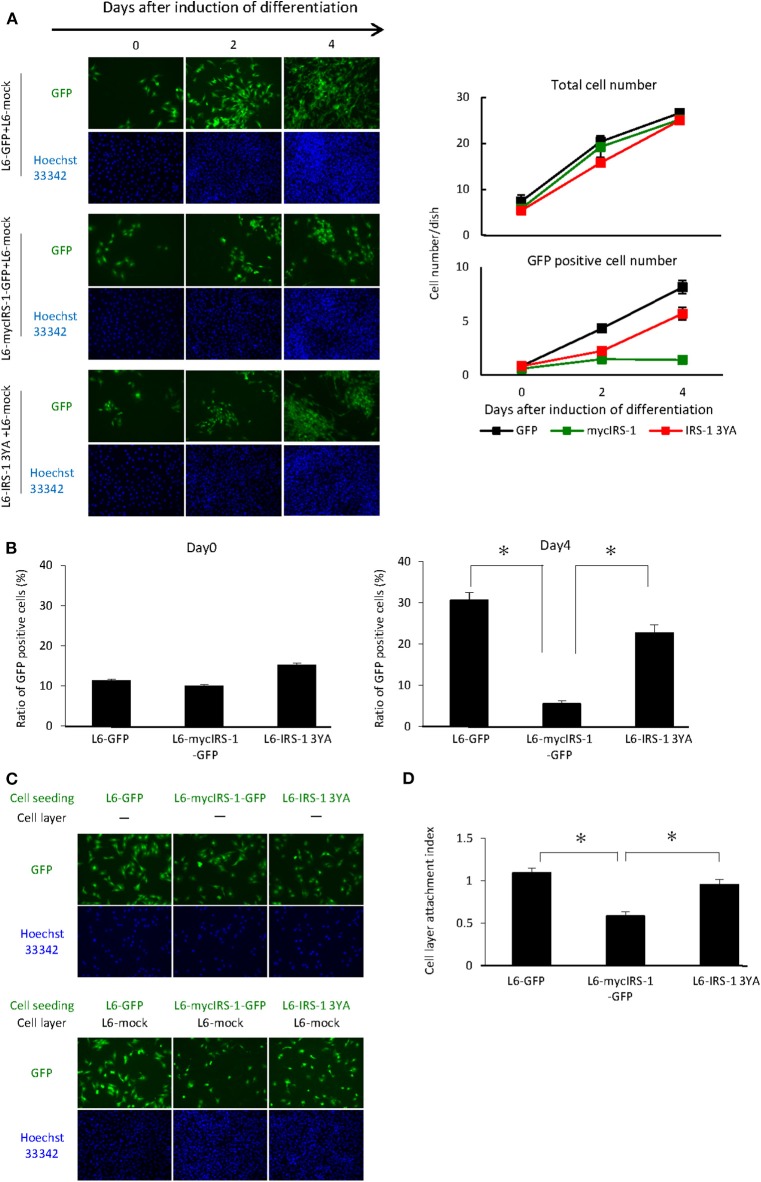
Elimination of cells overexpressing IRS-1 3YA mutant. **(A)** L6-GFP, L6-mycIRS-1-GFP, or L6-IRS-1-3YA cells were co-cultured with L6-mock in differentiation medium at a 1:10 ratio. At the indicated days after differentiation induction, cells were fixed by PFA, and the total nucleus numbers and the nucleus numbers of GFP positive cells were counted (right graphs). **(B)** The percentage of GFP-positive cells was calculated at day 0 or 4 after differentiation induction, as shown in the graph. Data is shown as means ± SEM. *p < 0.05 vs. day 0. **(C)** Five million L6-mock cells were seeded on the dish and cultured until confluent. L6-mycIRS-1-GFP, L6-GFP, or IRS-1 3YA mutant cells were then seeded on the L6-mock confluent cell layer or on a dish. After 1 day of incubation, the cells were fixed and the numbers of GFP-positive cells were counted. **(D)** CLAI was calculated as previously indicated, and data are shown as means ± SEM (*n* = 3), *p < 0.05. These are representative data from experiments independently performed at least three times.

As shown in Figure 2, we showed that cells with higher levels of IRS-1 were selectively eliminated from the cell layer when cultured with normal cells. Recently, we reported that IGF-I receptor internalization was inhibited in L6 myoblasts with high IRS-1 levels, resulting in sustained activation of IGF signaling. In addition, we prepared L6 myoblasts stably expressing IRS-1 3YA mutant and GFP (L6-IRS-1 3YA), which did not sustain IGF signal activation since this mutant could not inhibit internalization of the IGF-I receptor (20). In L6-IRS-1 3YA, differentiation-induced Caspase 3 activation was not enhanced (Figure S1), and the growth rate was almost identical with normal cells (Figure S2). Next, L6-IRS-1 3YA was cultured with normal cells, and we tested cell elimination from the cell layer. As shown in Figure 4A, elimination from the cell layer was observed when L6-mycIRS-1-GFP was cultured with L6-mock, whereas this was not observed when L6-IRS-1 3YA was co-cultured with L6-mock (Figures 4A,B). Under this situation, the attachment ability of L6-IRS-1 3YA to L6-mock cells was unchanged (Figures 4C,D).

Finally, we performed the similar experiments under the growth medium. L6-GFP, L6-mycIRS-1-GFP or L6-IRS-1 3YA was cultured with L6-mock normal cells in the DMEM 10% FBS and counted the nucleus number at the indicated days. Growth rate until 2 days was very similar for all cell lines. L6-GFP and L6-IRS-1 3YA could increase cell number until 4 days whereas L6-mycIRS-1-GFP could not increase the cell number but decreased (Figure S3). This data indicated that cells overexpressing IRS-1 were eliminated from the cell layer also under growth condition.

## Discussion

In this study, we revealed that IRS-1 overexpressing cells are eliminated from the cell layer upon culturing with normal cells. A similar phenomenon, cell competition, was observed in *Drosophila* (21). Cell competition is a cell fitness sensing mechanism where a less fit cell is eliminated as a “loser” when surrounded by fitter cells, or “winners.” In *Drosophila* imaginal discs, several mutants affecting cell proliferation including *Minute*, which have mutations in ribosomal genes, were reported to cause cell competition. Furthermore, cells with additional copies of *Myc* become “super-competitors” and can eliminate neighboring wild-type cells. Cells with mutant tumor suppressor genes *scribble* (*scrib*), *lethal giant larvae* (*lgl*), and *discs large* (*dlg*) lose cell polarity and are eliminated by the surrounding normal epithelial cells. In addition, recently, a Hippo signaling mutant caused cell competition in mammalian cells (22). Mammalian cells overexpressing active YAP1, which is a downstream protein of the Hippo signal, exhibit a cell-autonomous decrease in cell adhesion, and cell attachment to the culture dish influences the win-or-lose outcome of the competition with wild-type cells (23). In our L6 myogenic differentiation system, IRS-1-overexpressing cells are eliminated from the cell layer as loser cells. This is the first report that an IGF signaling protein was identified as a molecule that caused cell competition.

We have shown that a differential level of IRS-1 caused cell competition under a heterotypic population. However, we did not show that such cell competition could occur under physiological conditions. Recently, we reported the detailed molecular mechanism underlying the negative feedback loop of IGF signal transduction. In this paper, we present the precise mechanism of IGF-I-induced IRS-1 protein degradation. The downstream kinase mTORC1 phosphorylates the serine residue of IRS-1 at amino acid 422; this phosphorylation recruits ubiquitin ligase, resulting in IRS-1 degradation (19). Given our data, we envisage a possible scenario: since the IRS-1 protein level is dynamically changed in response to IGF stimulation and varies in each cell, cells with higher IRS-1 levels are eliminated under physiological conditions.

In this study, we have shown that attachment ability of IRS-1 overexpressing cells to L6-mock cells is lower than that of normal cells. These data suggest a possible mechanism of cell elimination of IRS-1 overexpressing cells. It is well-known that cells lose adhesion to dishes before cell division during proliferation. After cell division, divided cells invade into the cell layer again, but IRS-1 overexpressing cells cannot invade into the cell layer because of the low attachment ability to normal cells, resulting in selective elimination of IRS-1 overexpressing cells. Next question is “Do loser cells die?”. Our data showed that IRS-1 overexpressing cells are specifically eliminated from the cell layer. And at this differentiation stage, apoptotic cells increased, suggesting that eliminated loser cells might die by apoptosis. In Drosophila, apoptotic signal is activated in loser cells, including the JNK pathway. But in our model, to identify the apoptotic pathway was required for the further evaluation.

In order for the loser cells to be recognized by the winner cells, some components that label cells as “losers” are required. However, since IRS-1 is an intracellular protein, it could not be the candidate component of direct recognition for cell competition. Recently, we showed that IRS-1 interacts with the medium chain of clathrin-coated adaptor protein (AP2), and this interaction inhibited AP2 function to facilitate ligand-induced IGF-I receptor internalization. Thus, IRS-1 overexpression inhibited IGF-I receptor internalization, resulting in sustained Akt/mTORC1 activation (20). In this study, overexpression of IRS-1 mutant 3YA, which is an AP2-binding-deficient mutant, did not delay AP2-mediated IGF-IR endocytosis after ligand stimulation and did not cause cell competition. These data suggested that the accumulation of active IGF-I receptors on the plasma membrane labels cells as the loser cells. In addition, we showed that cell attachment to normal cells is inhibited in cells highly expressing IRS-1. It was reported that integrin and cadherin were bound to IRS-1 and the IGF-I receptor (24–26), and these molecules play roles in cell-cell or cell-extracellular matrix binding (27, 28). Moreover, Canonici et al. explained that IGF-I modulates association between IGF-I receptor, αv integrin and E-cadherin (29). These reports suggested that integrin and cadherin could be candidate components for labeling cells as the “losers.”

What is the relationship between cell competition and myogenic differentiation? Some reports demonstrated that apoptosis induced by the induction of myogenic differentiation was required for myoblast cell fusion (13). We expect that apoptosis induced by cell competition is also required for myoblast differentiation. Actually, we have reported that IRS-1 overexpression inhibited myogenic differentiation, and also demonstrated that continuous inhibition of Foxo1 due to sustained Akt activation caused defect of myogenesis possibly through repression of MyHC expression in IRS-1 overexpressing cells at the late stage of the differentiation process (12). These strongly suggested that defect of myogenesis in IRS-1 overexpressing cells was not only caused by disturbance of cell competition which happened in the beginning of differentiation. Cell competition might be a possible mechanism to prevent the subpopulation of myoblasts with high-level of IRS-1 from differentiating into myotubes. However, further analysis is required to evaluate involvement of cell competition in myogenic differentiation.

Why was cell competition induced by differential IRS-1 protein levels in myoblast cells? Skeletal muscle differentiation entails the coordination of muscle-specific gene expression and terminal withdrawal from the cell cycle, inducing permanent G1 phase. The execution of this pathway is required for the formation of multinucleated myotubes (30–33). These findings suggested that the cell cycle of myoblasts that were fusing to myotubes was adjusted to the G1 phase. It is well-known that in a variety of muscle types, IGF-I regulates proliferation through its effects on the cell cycle (34, 35). Furthermore, it was recently revealed that apoptotic myoblasts enhanced fusion (13). Based on these papers, we propose the hypothesis that cell competition monitored by the IRS-1 level induces apoptotic cells, in which the IGF signal and cell cycle phase differ from neighboring cells. Then, in the surviving cells, the IGF signal and cell cycle phase are easily synchronized, and these synchronized cells fuse to myotubes. Thus, it is possible that cell competition during myogenesis plays important roles for the functionally synchronized cells to fuse to myotubes.

We showed that myoblasts expressing high IRS-1 levels were eliminated upon culturing with normal cells. Furthermore, the sustained activity of the IGF-I receptor on the plasma membrane might be the signal for the loser cells. This mechanism can explain why cell proliferation and cell apoptosis can be induced at the same period during myogenic differentiation. We also found that the decrease in IRS-1 was also induced in adipogenesis (unpublished data). These results suggested that the decreases in IRS-1 and IGF signaling by IGF-I receptor downregulation could be crucial for cell differentiation.

L6 myoblasts that highly expressed IRS-1 protein were eliminated from the cell layer upon culturing with normal cells due to sustained activation of the IGF-I receptor on the plasma membrane. It is possible that cell competition induced by the differential level of IRS-1 is required for myogenic differentiation.

## Data Availability Statement

The datasets generated for this study are available on request to the corresponding author.

## Author Contributions

FH contributed to the conception and design of the study. RO and AU performed the experiments. S-IT wrote the manuscript. All authors discussed the results and approved the manuscript.

### Conflict of Interest

The authors declare that the research was conducted in the absence of any commercial or financial relationships that could be construed as a potential conflict of interest.
