# Supplementary material for: Myoblasts With Higher IRS-1 Levels Are Eliminated From the Normal Cell Layer During Differentiation
Source: Front Endocrinol (Lausanne). 2020 Feb 28;11:96. doi: 10.3389/fendo.2020.00096 (PMC7059307; doi:10.3389/fendo.2020.00096)
Supplement: Supplementary file 1 [file Data_Sheet_1.PDF]

Figure S1

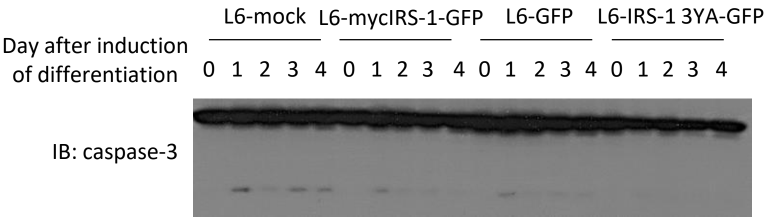

Figure S1. Apoptosis induced by differentiation in each L6 cell line
Differentiation of each L6 cell lines was induced by changing media from DMEM with 10% FBS to DMEM with 2% FBS. At the indicated days after differentiation induction, cell lysates were prepared, and total cell lysates were carried out for immunoblotting analysis using the indicated antibodies.

Figure S2

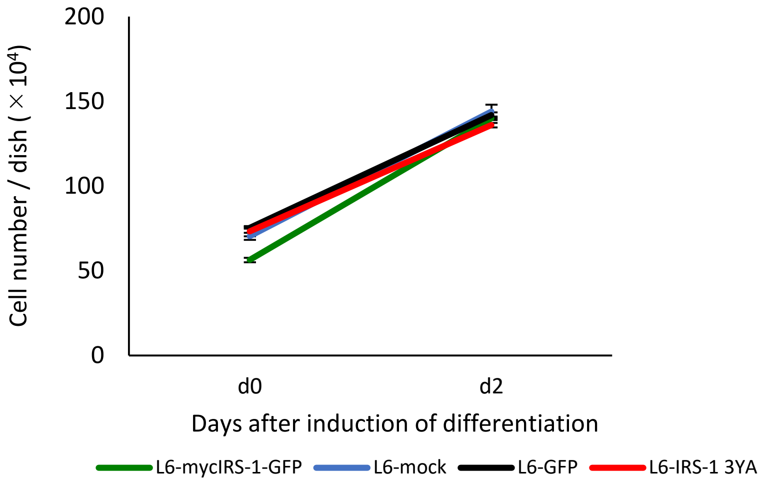

Figure S2. Proliferation rate of each cell line
L6-GFP, L6-mock, L6-mycIRS-1-GFP and L6-IRS-1 3YA were inoculated into dishes (1.0×10^5 cells/dish), and cultured. At the indicated days after differentiation induction, cell number was counted. The number of each cell was shown in the graph. Data is shown as means ± SEM.

Figure S3

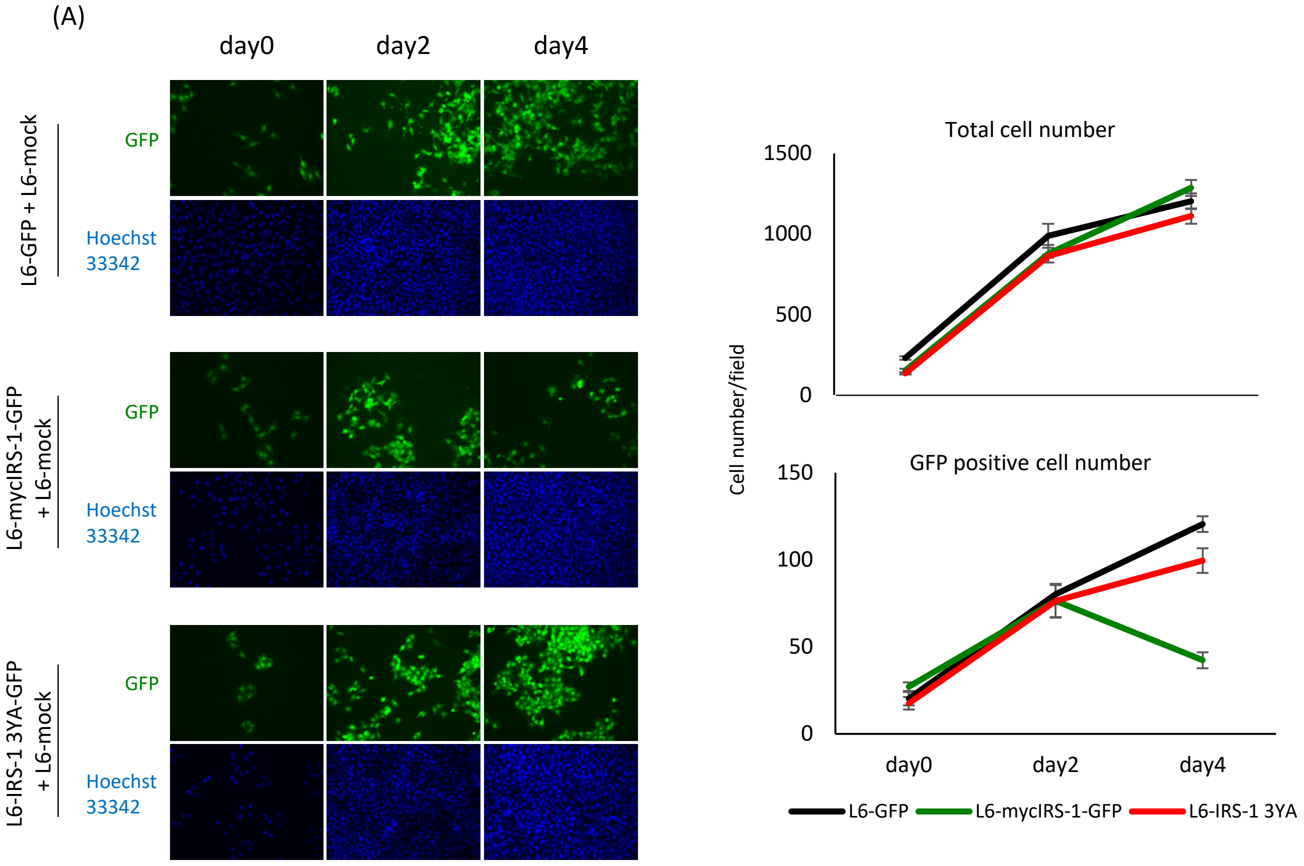

Figure S3. Elimination of cells under growth medium

L6-GFP, L6-mycIRS-1-GFP, or L6-IRS-1-3YA cells were cultured with L6-mock in the growth medium, DMEM with 10 % FBS, at a 1:10 ratio. At the indicated days, cells were fixed by PFA, and total nucleus numbers and the nucleus number of GFP-positive cells were counted (right graphs). Data is shown as means ± SEM.
